# Deep eutectic solvent extraction of chlorogenic acid from dandelion with ultrasonic-assisted: Process optimization, purification, and bioactivity

**DOI:** 10.1016/j.ultsonch.2025.107579

**Published:** 2025-09-27

**Authors:** Junhai Liu, Huan Duan, Hansheng Wang, Qi Gao, Lun Liu, Yinku Liang, Min He, Le Xu, Xiaosha Guo

**Affiliations:** a Qinba State Key Laboratory of Biological Resources and Ecological Environment (Incubation), School of Chemistry and Environment Science, Shaanxi University of Technology, Hanzhong 723001, PR China; b Hanzhong Shimen Solid Waste Disposal Co., Ltd, Hanzhong 723001, PR China; c Hanzhong Natural Gas Investment and Development Co., Ltd, Hanzhong 723001, PR China

**Keywords:** Deep eutectic solvent, Ultrasonic-assisted extraction, Dandelion, Chlorogenic acid, Antioxidant and antibacterial

## Abstract

Dandelion, a widely distributed medicinal plant, is rich in chlorogenic acid (CGA). However, research on the extraction and purification of CGA from dandelion, as well as its bioactivity, is insufficient. Therefore, this study developed an efficient and economic method for extracting CGA from dandelions using ultrasonic-assisted deep eutectic solvents. The DES system comprised betaine and oxalic acid, demonstrated high extraction efficiency, and was identified as the optimal extraction solvent. Furthermore, Response Surface Methodology (RSM) was employed to optimize the extraction conditions, which included: water content of 42 %, ultrasonic power of 300 W, ultrasonic temperature of 70 °C, ultrasonic time of 32 min, and solid–liquid ratio of 1:30 g/mL. Under these conditions, the extraction yield of CGA reached 4.27 mg/g. Dandelion samples treated with ultrasound-assisted DES had improved extraction efficiency. Following further purification with macroporous resin, the purified extract of CGA (PECGA) demonstrated significant antioxidant activity. Moreover, this ultrasonic-assisted PECGA showed considerable antibacterial efficacy against *Staphylococcus aureus* (*S. aureus*) and *Escherichia coli* (*E. coli*). This study provides evidence for potential applications of CGA extracted from dandelions as a natural antioxidant and antibacterial agent in the food and pharmaceutical industries.

## Graphical abstract

**Figure:**
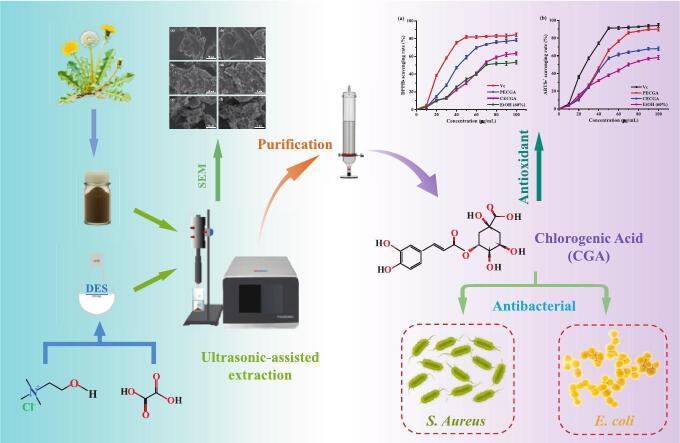

## 1 Introduction

Dandelion (*Taraxacum officinale*) is a perennial herbaceous plant that belongs to the family asteraceae [1]. It is rich in bioactive compounds, including chlorogenic acid (CGA), caffeic acid, and luteolin. Furthermore, it has significant biological activities, such as antioxidant and antibacterial effects [2]. Because of these properties, dandelion is considered a promising candidate for developing novel pharmaceuticals and functional foods [3]. CGA is a water-soluble phenolic compound with diverse beneficial functions, such as antimicrobial, antioxidant, antihypertensive, and anti-inflammatory effects [[4], [5], [6], [7]]. Therefore, it has gained significant attention as a natural food preservative, antioxidant, and colorant [8,9]. However, its inherent polarity and sensitivity to hydrolysis and esterification require the improvement of extraction procedures to maximize their extraction yield [[10], [11], [12]].

Various CGA extraction methods have been investigated, such as water extraction [13], organic solvent extraction [14], ultrasonic-assisted extraction [15], microwave-assisted extraction [16], and enzymatic methods [17]. The hot water extraction technology is eco-friendly and economical; however, its efficiency constraints limit widespread implementation. With advancements in science and technology, the CGA extraction process has undergone iterative innovations [13,18]. The mechanism underlying ultrasonic-assisted extraction involves acoustic cavitation, which generates localized high shear forces, micro-jets, and shockwaves that disrupt cell walls and enhance mass transfer [19]. Compared to the traditional hot water extraction method, ultrasonic-assisted extraction combines the advantages of easy operation, lower energy consumption, and higher extraction efficiency within a shorter time [20,21]. It is widely used in the food, chemical, metallurgy, environmental, and other fields [22,23]. However, the current single extraction procedure does not yield the expected outcomes and warrants further research on efficient and eco-friendly alternatives for CGA extraction.

Deep eutectic solvents (DES) are innovative solvents typically composed of a hydrogen bond acceptor (HBA) with one or more hydrogen bond donors (HBD) [13,[24], [25], [26]]. Relative to traditional solvent systems, DES is cost-effective, easy to synthesize, low or non-toxic, biocompatible, and recyclable [21,27,28]. Furthermore, it has improved the extraction yield, purity, and biological activity of CGA extract [29]. For instance, Jiang et al. [30] reported a CGA extraction yield of 4.36 mg/g from Eucommia leaves using a 50 % ethanol solution as a solvent. Yue et al. [31] established a method that combined solvent effect theory and chemical calculations to guide DES selection for CGA extraction. They revealed that proline-malic acid was the optimal solvent, achieving the highest CGA yield of 3.77 mg/g in the tested DES. These findings underscore the significance of DES in CGA extraction. Therefore, the synergistic integration of ultrasonic-assisted extraction with DES has been proposed as an efficient technique for the isolation and purification of CGA, and presents a promising green extraction strategy.

The antioxidant activity of CGA is significant for the maintenance of the human health system; it can effectively scavenge harmful free radicals from the body and avoid oxidative attacks on biological macromolecules [32,33]. Increased oxidative stress has been associated with chronic conditions, including neurodegenerative diseases, cardiovascular diseases, and diabetes [29]. Therefore, investigating efficient and safe antioxidant strategies for CGA has become crucial for the scientific and medical communities. Antioxidants can be categorized into synthetic and natural sources according to their origin. Although synthetic antioxidants may provide possible toxicity and undesirable effects, natural antioxidants are generally preferred [[34], [35], [36]]. CGA, a significant component of plant secondary metabolites, has exceptional free radical scavenging capabilities and antioxidant properties attributable to its abundant phenolic hydroxyl groups. This compound not only decelerates the aging process in organisms but also plays a multifaceted role in disease prevention, indicating wide application potential in functional food and medicinal domains [37,38].

**Fig. 1:**
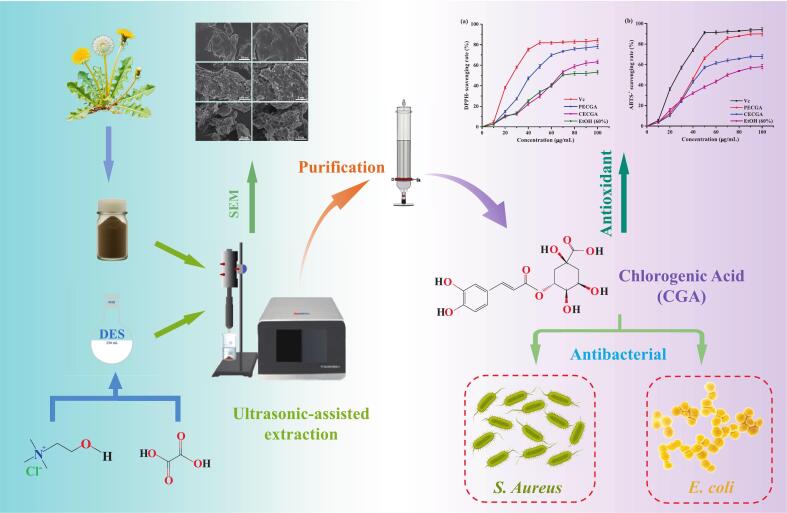
Schematic overview of the extraction, purification, and evaluation of antioxidant and antibacterial properties of CGA from dandelion.

Therefore, as shown in Fig. 1, in this study, a combination of DES of 20 species was selected for the ultrasonic-assisted CGA extraction from dandelion. The extraction process was further optimized using single-factor experiments and Box-Behnken Design (BBD) response surface methodology. The optimized extraction conditions identified included water content of 42 %, ultrasonic power of 300 W, ultrasonic temperature of 70 °C, ultrasonic time of 32 min, and solid–liquid ratio of 1:30 g/mL, which extract yield about 4.27 mg/g. Similarly, the crude extract of chlorogenic acid (CECGA) obtained was purified using NKA-II. Furthermore, the in vitro antioxidant and bacteriostatic properties of the purified extract were investigated. The findings will provide an efficient and economic method for CGA extraction and purification from dandelion, as well as provide evidence for its potential as a valuable natural product.

## 2 Materials and methods

### 2.1 Materials and reagents

Ethanol (EtOH), Phosphoric acid (PA), Betaine (Bet), Oxalic acid (Oxal), Citric acid (Cit), Lactic acid (LA), Glycerol (Gly), Ethylene glycol (EG), Sucrose (Suc), D-glucose (Glc), D-Fructose (Fru), Xylitol (Xyl), Urea (U), DL-Malic acid (L-MA), Choline chloride (ChCl), Vitamin C (Vc) were of analytical grade and acquired from Sinopharm Chemical Reagent Co., Ltd. Acetonitrile (≥99.5 %, GC), CGA standard (≥98 %) were purchased from Shanghai Aladdin Biochemical Technology Co., Ltd. Ascorbic acid were obtained from Sichuan Yike Pharmaceutical Co., Ltd. 2,2′-azinobis (3-ethylbenzothiazoline-6-sulfonic acid ammonium salt) (ABTS) and 1,1-diphenyl-2-picrylhydrazyl (DPPH) were acquired from Nanjing Yuanzhi Biotechnology Co., Ltd. Luria Bertani (LB) Broth and agar were provided by Beijing Solarbio Science & Technology Co., Ltd. *Staphylococcus aureus* (*S. aureus*) and *Escherichia coli* (*E. coli*) were obtained from Beijing Zhongke Quality Inspection Biotechnology Co., Ltd. KBC-500F split ultrasonic processor, Kunshan Ultrasonic Instruments Co., Ltd. Cary 50 UV–Vis Spectrophotometer, Varian, U.S.A. LC-2030 High Performance Liquid Chromatograph, Shimadzu, Japan. Dandelion was harvested from Hanzhong City (Shaanxi Province, China), cleaned, dried at a constant 50°C temperature until constant weight was acquired, and then crushed, passed through a 40-mesh sieve, and set aside.

### 2.2 Preparation of deep eutectic solvents

Based on previous literature on the extraction of phenolic compounds from plants [[39], [40], [41]], hydrophilic DES was selected for CGA extraction. DES were prepared by mixing 2 HBA (including ChCl and bet) and 11 HBD. Furthermore, the molar ratio of HBD to HBA was adjusted to prepare 20 different DES systems. The quantities of HBA and HBD were accurately measured and added to 250 mL round-bottom flasks. Deionized water, constituting 40 % (w/w) of the combined mass of HBA and HBD, was then added to reduce the viscosity of the DES. The solution was agitated in a water bath at 80 °C until it became transparent. The formulations of the 20 prepared DES are listed in Table 1 [[42], [43], [44]].

**Table 1:** Preparation of DES.

No.	HBA	HBD	Mole ratio
DES-1	Bet	Oxal	1:1
DES-2	Bet	Cit	1:1
DES-3	Bet	LA	1:2
DES-4	Bet	Gly	1:2
DES-5	Bet	EG	1:1
DES-6	Bet	Suc	1:2
DES-7	Bet	Glc	1:1
DES-8	Bet	Fru	1:2
DES-9	Bet	Xyl	1:1
DES-10	Bet	U	1:2
DES-11	ChCl	Oxal	1:1
DES-12	ChCl	Cit	1:1
DES-13	ChCl	LA	1:2
DES-14	ChCl	Gly	1:2
DES-15	ChCl	EG	1:1
DES-16	ChCl	Suc	1:1
DES-17	ChCl	Glc	1:1
DES-18	ChCl	Mal	1:1
DES-19	ChCl	Xyl	1:1
DES-20	ChCl	U	1:2

### 2.3 CGA extraction and DES screening

Dandelion powder (1.00 g) was added to a 50 mL centrifuge tube and mixed with DES solvent. Then, the ultrasonic extraction time, temperature, and power were set. The CGA was then extracted from dandelion to obtain CECGA, followed by its quantification and extraction yield (mg/g) calculation.

For subsequent experiments, the types of DES were screened by setting the DES water content to 40 %, the ultrasonic time to 30 min, the ultrasonic temperature to 60 °C, the ultrasonic power to 300 W, and the solid–liquid ratio to 1:40 g/mL. A 60 % (V/V) aqueous solution of EtOH was used as a parallel control group for ultrasonic extraction under the same conditions.

### 2.4 Single-factor experiment

The single-factor experiment was performed by following the method of Liu et al. [45,46] to investigate CGA extraction yield under different extraction conditions. During optimization, each extraction parameter was set to five different levels within a certain range: DES water content of 10, 20, 30, 40, and 50 %, ultrasonic time of 10, 20, 30, 40, and 50 min, ultrasonic temperature of 50, 60, 70, 80, and 90 °C, ultrasonic power of 100, 200, 300, 400, and 500 W, solid–liquid ratio of 1:20, 1:30, 1:40, 1:50, and 1:60 g/mL.

### 2.5 Response surface experiment

To optimize the CGA extraction process and achieve the highest yield, based on the results of single-factor experiments, the Orthogonal Central Composite Design (OCCD) of RSM was employed to investigate the association between various factors. The primary factors and their respective levels considered are indicated in Table S1 and include DES water content A (%), ultrasonic power B (W), ultrasonic temperature C (°C), ultrasonic time D (min), and solid–liquid ratio E (g/mL).

### 2.6 Determination of CGA from dandelion

Briefly, the extract was diluted with distilled water to a total volume of 100 mL and subsequently filtered through a 0.45 µm membrane before analysis. The requirements for HPLC were as follows: column of C8-3, mobile phase of acetonitrile +0.2 % aqueous phosphoric acid (15:85, V/V), flow rate of 1 mL/min, detection wavelength of 330 nm, injection volume of 5 µL, and column temperature of 30 °C. The concentration of CGA was determined using a standard curve.

### 2.7 Scanning electron microscopy (SEM) analysis

The solid morphology of the raw and ultrasonically-treated dandelion powders was assessed by following a previously described method [47]. The samples were then photographed and analyzed by gold spraying using a vacuum sputtering apparatus, followed by SEM (Regulus 8100, Japan Hitachi) under the accelerated condition of 20 kV [48].

### 2.8 Dandelion CGA adsorption purification

#### 2.8.1 Pretreatment of macroporous resin

In this investigation, five varieties of macroporous resins (HPD826, D101, AB-8, NKA-II, and NKA-9) were introduced into a glass chromatographic column to guarantee that the height-to-diameter ratio of the column bed surpassed 5:1. Then, the resin was immersed in EtOH for 24 h and rinsed thoroughly multiple times with distilled water until the EtOH odor was gone. The resin was submerged in a 3 % dilute hydrochloric acid solution for 2 h, subsequently washed with distilled water until the wash solution attained a neutral pH. The resin was immersed in a 3 % sodium hydroxide solution for 4 h and rinsed with distilled water until the effluent was neutral.

#### 2.8.2 Screening of macroporous resins

Each pretreated macroporous resin was added to a 150 mL stoppered conical flask and mixed with 20 mL CECGA. The conical flasks were shaken for 6 h at 35 °C and 150 rpm, in the dark, for adsorption. At the end of the adsorption, the supernatant was aspirated and filtered. The CGA concentration was determined. The adsorption rate and adsorption capacity were calculated according to equations (1), (2).

(1) $$Adsorptionrate\left(\%\right)=\frac{B_{0}-B_{1}}{B_{0}}\times 100$$

(2) $$Adsorptioncapacity\left(\mathrm{mg}/g\right)=\frac{B_{0}\;\text{-}\;B_{1}}{M}\times V$$

The five adsorbed resins were aspirated, filtered, and subsequently transferred to a 150 mL stoppered conical flask. Desorption was accomplished by introducing 20 mL of an EtOH solution and agitating the mixture at 35 °C and 150 rpm in the dark for 6 h. The supernatant was subsequently aspirated and filtered, and the concentration of CGA was determined. The desorption rates of the different resins used for cattail CGA were calculated by Equation (3).

(3) $$Desorptionrate\left(\%\right)=\frac{B}{B_{0}-B_{1}}\times 100$$

where, *B*_0_ = the concentration of CECGA in mg/mL, *V* = the volume of CECGA in mL, *B*_1_ = the concentration of CGA in mg/mL, and *M* = pretreated macroporous resin in g.

#### 2.8.3 Optimization of adsorption and desorption conditions

Pretreated NKA-II macroporous resin (20.0 g) was weighed, and the column was loaded by the wet method. After CECGA adsorption, desorption was performed using EtOH. The effluent was collected in 10 mL increments, and the concentration of CGA was measured. The effects of adsorption rate, desorption rate, and desorption volume on the adsorption and desorption of CGA were studied, respectively. All parameters were set as follows: adsorption rate at 1, 2, 3, 4, and 5 BV/h; desorption rate at 1, 2, 3, 4, and 5 BV/h; and desorption volume at 1, 2, 3, 4, and 5 BV.

### 2.9 Antioxidant experiment

CECGA, PECGA, and Vc solutions were prepared at concentrations of 1.0, 2.0, 3.0, 4.0, and 5.0 μg/mL, where Vc was used as a control. Then, 3.0 mL of CECGA, PECGA, and Vc were mixed with 3.0 mL DPPH solution at a 0.1 mmol/L concentration. The reaction was performed for 30 min in a water bath at 30 °C in the dark, and the absorbance (*A*_1_) was recorded at 517 nm upon completion of the reaction. Furthermore, 3.0 mL of CECGA, PECGA, and Vc were combined with 3.0 mL of anhydrous ethanol. The reaction parameters were identical to DPPH, and the absorbance (*A*_2_) was recorded at 517 nm. Moreover, 3.0 mL of DPPH solution, at a concentration of 0.1 mmol/L, was combined with 3.0 mL of a 50 % ethanol aqueous solution (V/V) with the same reaction conditions as above. The absorbance (*A*_0_) was measured at 517 nm. Finally, the clearance rate was calculated according to equation (4):

*SR*_DPPH•_ = ×100 % (4).

The ABTS·+ clearance was assessed using the same procedure as the DPPH· assay, with the detection wavelength set at 734 nm.

### 2.10 Antibacterial analysis

#### 2.10.1 Preparation of culture media

The LB liquid medium was prepared by mixing 2.5 g of LB broth powder and 100 mL of distilled water. It was then sterilized in an autoclave at 121 °C for 15 min and cooled before use.

The LB solid medium was prepared by mixing 2.5 g of LB broth medium and 1.5 g of agar powder into a 250 mL conical flask. Then, 100 mL of distilled water was added and mixed thoroughly. The mixture was then sterilized for 15 min in a sterilization kettle at 121 °C and cooled to approximately 40–50 °C. Lastly, 15 mL of the medium was poured into a disposable aseptic petri dish under aseptic conditions.

#### 2.10.2 Preparation of bacterial suspension

The LB liquid medium (3 mL) was poured into three 12 mL test tubes, respectively. Then, single colonies of *S. aureus* and *E. coli* were selected from the solid medium and inoculated into two test tubes. The third tube was used as a blank control. The tubes were incubated for 15 h in a constant temperature shaker at 37 °C and 200 rpm.

#### 2.10.3 Preparation of samples for testing

Briefly, PECGA (250 mg) that had been UV sterilized for 30 min was mixed with 1 mL of DMSO to prepare a 250 mg/mL PECGA mother liquor. The solution was then filtered through a 0.22 μm filter to remove bacteria, and diluted to a 50 mg/mL sample-medium mixture using LB liquid medium. Both bacterial solutions were diluted to 10^6^ CFU/mL as well as 5-fold in DMSO using LB liquid medium, respectively. Then, three parallel experiments were performed with control group (1) (DMSO-LB liquid medium + bacterial solution) and control group (2) (DMSO-LB liquid medium).

#### 2.10.4 Minimal inhibition concentration (MIC) analysis

Based on the previous methods [48], LB liquid medium (100 μL/well) was added to 2 to 12 wells of the 96-well plate. In the 1st well, 200 μL of CGA medium mixture (128 mg/mL) was added. Subsequently, 100 μL of the mixture was pipetted from well 1 and transferred to well 2, and thoroughly mixed. 100 μL of the mixture was pipetted from well 2 and transferred to well 3, and thoroughly mixed. This process of dilution continued sequentially up to well 12, from which 100 μL of the mixture was discarded. Rows G and H were set as controls, contained 20 % DMSO-LB liquid medium instead of the PECGA solution, and were also serially diluted. Furthermore, 100 μL of a diluted bacterial solution (initial concentration of 25 mg/mL) was added to each well in the sample rows A, B, and C, as well as to the control row of G. Moreover, 100 μL of LB liquid medium was added to each well in the control row of H. The sample rows of A, B, and C, along with the control row of G, were treated uniformly. The 96-well plates were placed in a constant-temperature incubator at 37 °C for 24 h and then photographed. The minimum sample concentration for culture medium clarification is the MIC. Taking photos and recording the MIC.

## 3 Results and discussion

### 3.1 DES screening

**Fig. 2:**
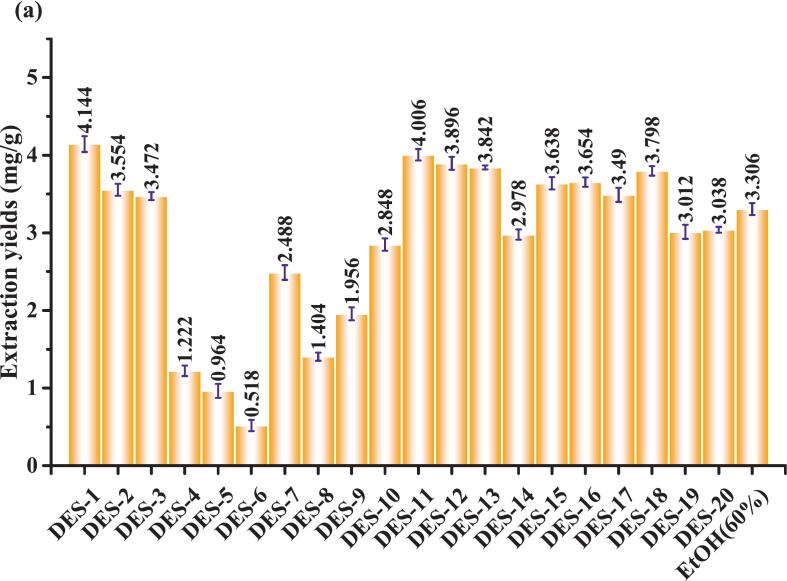
DES screening and selection for CGA extraction.

The results showed that CGA extraction using DES prepared with sugars and alcohols as HBD was low (Fig. 2), whereas that using DES formulated with organic acids as HBD was significantly higher, particularly when oxalic acid was employed as the HBD, achieving the highest extraction yields of 5.034 and 4.928 mg/g, respectively. Furthermore, 8 DES extractions yielded results exceeding 60 % EtOH (extraction: 4.276 mg/g). A betaine-oxalic acid mixture in a 1:1 ratio was selected as the DES for CGA extraction from dandelion. This choice was guided by both physicochemical and environmental considerations. Betaine is highly water-soluble and forms stable hydrogen bonds with organic acid–based HBDs. Oxalic acid, as a dibasic carboxylic acid, provides multiple donor sites that align with the polarity of CGA, improving solubility. At 40 % water content, the DES maintains moderate viscosity, supporting a stable hydrogen-bonding network while facilitating ultrasonic cavitation. This accelerates cell wall disruption and improves mass transfer efficiency. Moreover, both betaine and oxalic acid are biodegradable and of low toxicity, aligning with the principles of green and safe extraction. Collectively, these advantages identified betaine–oxalic acid DES as the most suitable solvent system for subsequent experiments [19,22,23].

### 3.2 Effect of a single factor on CGA yields

**Fig. 3:**
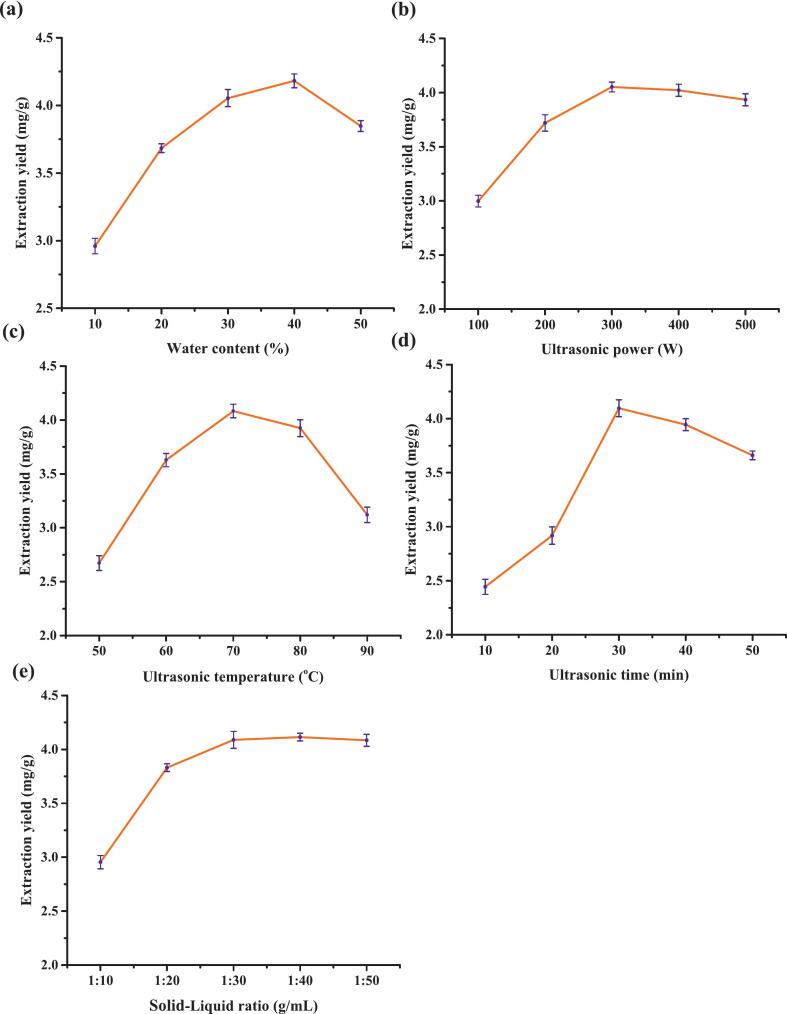
Effect of (a) water content, (b) ultrasonic power, (c) ultrasonic temperature, (d) ultrasonic time, and (e) solid–liquid ratio on CGA extraction yield.

To investigate the effect of the water content in DES on the extraction yield of CGA, a series of experiments was performed with a gradient range of 10–50 %, while other parameters remained the same, including ultrasonic time of 30 min, ultrasonic temperature of 70 °C, ultrasonic power of 300 W, and solid–liquid ratio of 1:40 g/mL. The results showed that as the water content increased from 10 to 40 %, the extraction yield of CGA increased significantly, peaking at 40 % (Fig. 3a). However, when the water content was further increased to between 40 % and 50 %, the extraction yield declined. This may be because the addition of water reduces DES viscosity and weakens the strength of hydrogen bonding, thus increasing molecular mobility and facilitating the mass transfer of CGA. However, excessive water can disrupt the internal hydrogen bonding network of DES, weakening their selective solubility and potentially diluting the DES structure, reducing the extraction efficiency [44,49,50]. Furthermore, excessively elevated water content may facilitate the co-solubilization of contaminants, exerting a competitive inhibitory impact on the target molecules. Therefore, considering the solvent economy and extraction efficiency, a water content of 40 % was selected for the remaining experiments.

The ultrasonic power was experimentally examined in the 100–500 W range (keeping the remaining criteria the same). It was observed that when the power was increased from 100 W to 300 W, the CGA extraction yield increased significantly. However, upon further increase to 400 W, no change was observed (Fig. 3b). Insufficient cavitation at low power leads to incomplete cell fragmentation, whereas excessively high power only marginally increases the extraction yield and may instead cause degradation of CGA due to localized overheating or the generation of free radicals [42,51,52]. Therefore, 300 W was selected as a cost-effective ultrasonic power.

To identify optimized ultrasonic temperature, serial experiments with temperatures ranging from 50 to 90 °C (remaining conditions constant as described above) were performed. The CGA extraction yield increased with rising temperature, reaching a maximum at 70 °C; however, it reduced with further increase in the temperature to 80 °C (Fig. 3c). This could be because moderate warming reduces DES viscosity and lowers the cavitation threshold, which increases cell wall rupture and accelerates mass transfer. However, CGA is thermally sensitive, and elevated temperatures accelerate its hydrolysis or oxidative degradation, reducing the mass transfer benefits associated with temperature [51,53,54]. Therefore, 70 °C was selected as the optimal temperature for ultrasonic extraction in the subsequent experiments.

The effect of ultrasonic time on CGA yield was assessed at different time points with other parameters constant as stated above. It was observed that during 10–30 min, the yield increased significantly over time, reaching a maximum value at 30 min. However, when the duration was extended to 50 min, the yield did not increase but decreased slightly (Fig. 3d). This might be attributed to a short extraction time, which makes it difficult to fully solubilize CGA, whereas an excessively long period of ultrasonic-heat synergy may degrade or oxidize CGA [51,52,55]. Further, prolonged cavitation may promote side reactions, resulting in the loss of target components. Thus, 30 min was selected as an appropriate extraction time to balance efficiency and energy consumption.

Lastly, the solid–liquid ratio experiments were conducted within the range of 1:10–1:50 g/mL, keeping the remaining conditions constant. The results showed that CGA yield increased with the rising solvent dosage and levelled off after a ratio of 1:40 g/mL (Fig. 3e). When the solid–liquid ratio exceeded 1:40 g/mL, further increasing the solvent volume failed to increase the extraction yield, promoted solvent wastage, and increased energy consumption for subsequent concentration processes. This might be because when the solvent volume reaches the mass transfer equilibrium point, the target component is completely dissolved, and any excess solvent merely dilutes the system without improving efficiency [56,57]. Therefore, a solid–liquid ratio of 1:30 g/mL was selected as the most suitable.

### 3.3 RSM analysis and discussion

The results of the response surface analysis were analyzed using SAS V8.1 software and are shown in Table 2. The ANOVA for factors is presented in Table S2, the ANOVA for equations is displayed in Table 3, and the ANOVA for response variables is shown in Table 4 [58].

**Table 2:** Experimental scheme and result of RSM.

RUN	A	B	C	D	E	Y	RUN	A	B	C	D	E	Y
1	−1	−1	−1	−1	1	3.21	19	0	−2	0	0	0	3.62
2	−1	−1	−1	1	−1	3.27	20	0	2	0	0	0	3.46
3	−1	−1	1	−1	−1	3.51	21	0	0	−2	0	0	3.54
4	−1	−1	1	1	1	3.67	22	0	0	2	0	0	3.51
5	−1	1	−1	−1	−1	3.50	23	0	0	0	−2	0	3.67
6	−1	1	−1	1	1	3.42	24	0	0	0	2	0	4.09
7	−1	1	1	−1	1	3.54	25	0	0	0	0	−2	3.65
8	−1	1	1	1	−1	4.14	26	0	0	0	0	2	3.55
9	1	−1	−1	−1	−1	3.48	27	0	0	0	0	0	4.20
10	1	−1	−1	1	1	3.76	28	0	0	0	0	0	4.19
11	1	−1	1	−1	1	3.85	29	0	0	0	0	0	4.21
12	1	−1	1	1	−1	3.64	30	0	0	0	0	0	4.17
13	1	1	−1	−1	1	3.76	31	0	0	0	0	0	4.32
14	1	1	−1	1	−1	3.89	32	0	0	0	0	0	4.30
15	1	1	1	−1	−1	3.34	33	0	0	0	0	0	4.29
16	1	1	1	1	1	3.32	34	0	0	0	0	0	4.31
17	−2	0	0	0	0	3.72	35	0	0	0	0	0	4.19
18	2	0	0	0	0	4.12	36	0	0	0	0	0	4.32

**Table 3:** Variance analysis of the regression equation.

Source	Degrees of freedom	Sum of squares of deviation	Mean square	F	Pr > F
Total model	20	4.2781	0.9754	29.74	<0.0001
Linear	5	0.2610	0.0595	7.26	0.0012
Quadratic	5	3.1952	0.7285	88.86	<0.0001
Crossproduct	10	0.8220	0.1874	11.43	<0.0001
Residual	15	0.1079	0.0072		
Lack of fit	6	0.0727	0.0121	3.10	0.0625
Pure error	9	0.0352	0.0039		
Total error	35	4.3860

**Table 4:** Variance analysis of experimental results.

Source	DF	SS	MS	F	Pr > F	Significant
A	1	0.1040	0.1040	14.4642	0.0017	**
B	1	0.0017	0.0017	0.2318	0.6372	
C	1	0.0182	0.0182	2.5239	0.1330	
D	1	0.1291	0.1291	17.9476	0.0007	**
E	1	0.0081	0.0081	1.1217	0.3063	
A*A	1	0.1943	0.1943	27.0149	0.0001	**
A*B	1	0.1156	0.1156	16.0750	0.0011	**
A*C	1	0.3025	0.3025	42.0647	0.0001	**
A*D	1	0.0196	0.0196	2.7255	0.1195	
A*E	1	0.0529	0.0529	7.3561	0.0161	*
B*B	1	0.9568	0.9568	133.0505	0.0001	**
B*C	1	0.0870	0.0870	12.1014	0.0034	**
B*D	1	0.0072	0.0072	1.0047	0.3321	
B*E	1	0.1260	0.1260	17.5247	0.0008	**
C*C	1	0.9988	0.9988	138.8839	0.0001	**
C*D	1	0.0012	0.0012	0.1703	0.6856	
C*E	1	0.0042	0.0042	0.5875	0.4553	
D*D	1	0.2473	0.2473	34.3942	0.0001	**
D*E	1	0.1056	0.1056	14.6879	0.0016	**
E*E	1	0.7980	0.7980	110.9682	0.0001	**
Model	20	4.2781	0.2139	29.7450	0.0001	**
Error	15	0.1079	0.0072			
Total	35	4.3860				

Note: * indicates significant, ** indicates highly significant.

A quadratic polynomial regression was applied to the experimental data to establish a model that correlates DES water content (A), ultrasonic power (B), ultrasonic temperature (C), ultrasonic time (D), solid–liquid ratio (E), and CGA yield (Y) as follows:

Y = 4.246944 + 0.065833*A + 0.008333*B + 0.0275*C + 0.073333*D − 0.018333*E − 0.077917*A*A − 0.085*A*B − 0.1375*A*C − 0.035*A*D + 0.0575*A*E − 0.172917*B*B − 0.07375*B*C + 0.02125*B*D − 0.08875*B*E − 0.176667*C*C + 0.00875*C*D − 0.01625*C*E − 0.087917*D*D − 0.08125*D*E − 0.157917*E*E.

The ANOVA results (Table 3) indicated that this quadratic regression model was highly significant (p < 0.0001) [59]. Furthermore, the misfit test showed that p = 0.0625 > 0.05, indicating that the model has a good fit, that is, no misfit factor exists. This factor can effectively elucidate the differences in the response value. The regression model can be employed instead of the actual experimental data point to predict and examine the experimental outcomes.

The magnitude of the coefficient of determination, R^2^, reflects the proportion of the variation in the dependent variable (response value) explained by the model's independent variable (factor). The adjustment of R^2^ (R^2^_adj_) accounts for the number of independent variables in the model. The model complexity analysis provides a more objective reflection of the model and prevents overfitting due to an excessive number of variables. A reduced disparity between the two enhances the model's simplicity and validity. The R^2^ was 97.54 %, indicating that the model explains 97.54 % of the variation in the response values. Whereas R^2^_adj_ was 94.26 %, which showed a strong correlation between the model and the selected variables. Despite the increased number of variables, the model did not have overfitting, demonstrating high reliability and predictive capability. The coefficient of variation (CV) of the model was 2.23 %, suggesting that the experimental design is good. The fluctuation of the experimental data was minimal, and the experimental results demonstrated high reliability and stability. This further validated the predictive power of the regression model and the effectiveness of controlling the experimental conditions.

**Fig. 4:**
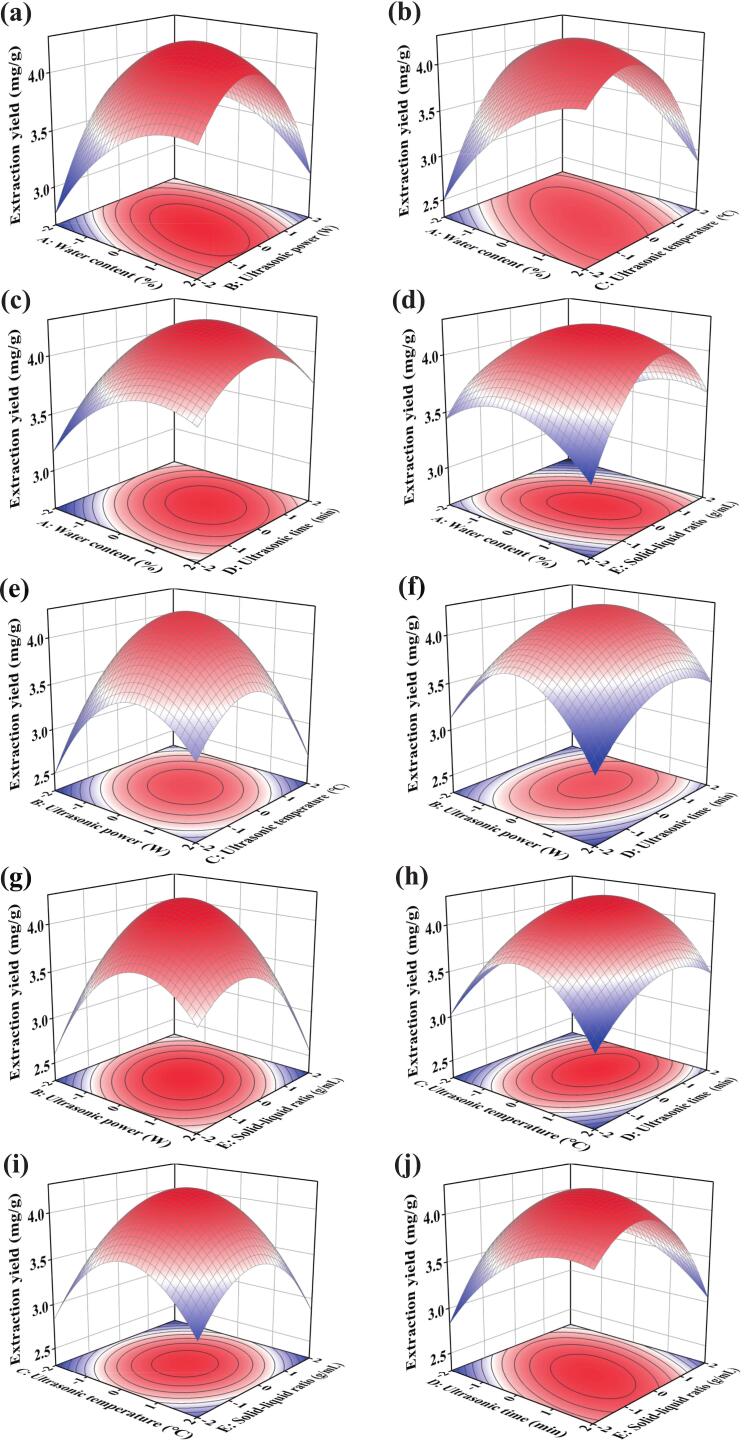
The effects of water content, ultrasonic power, ultrasonic temperature, ultrasonic time, and solid–liquid ratio on the CGA from dandelion. (a), (b), (c), (d), (e), (f), (g), (h), (i), and (j) indicate the response surface plots.

The interaction illustrates the synergistic impact on the response value when two factors function together. The A*E attained statistical significance (0.01 < p < 0.05) (Table 4), and the contour lines in Fig. 4d showed an elliptical shape. The interactions A*B, A*C, B*C, B*E, and D*E attained a statistically significant level (p < 0.01) (Fig. 4). This suggests that it is inadequate to only examine the effects of individual factors, and the interactions between the variables should be considered. Overall, it was inferred that when one variable alters, the other must be adjusted to optimize the extraction efficacy.

All five factors, A, B, C, D, and E, had highly significant effects on the extraction of CGA from dandelion (Table S2), suggesting that all these factors play a crucial role in the experiment, allowing the model to better fit the experimental data.

The predicted optimal conditions were A = 0.164667, B = 0.000124, C = −0.018049, D = 0.197493, and E = −0.048957, resulting in an optimal value of 4.2727. The optimal conditions were subjected to minor modifications to improve operational feasibility (Table S1). The final optimal conditions were as follows: water content of 42 %, ultrasonic power of 300 W, ultrasonic temperature of 70 °C, ultrasonic duration of 32 min, and solid–liquid ratio of 1:30 g/mL.

Based on the optimal conditions, 5 replicate validation experiments were performed, which revealed the average CGA extraction yield of 4.27 mg/g. The extraction yield exceeded the documented values of CGA extracted from different plants using various techniques (Table S3). The results validate that the regression model accurately depicts the experimental data and is appropriate for guiding an actual extraction procedure.

### 3.4 The results of SEM analysis

**Fig. 5:**
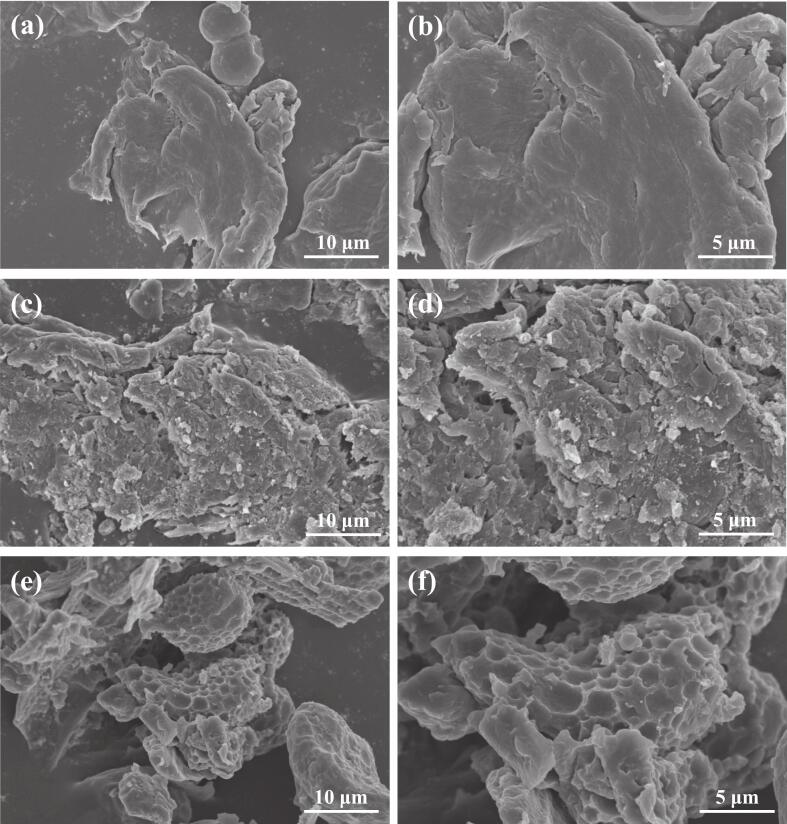
SEM micrographs of dandelion in (a, b) crude form, (c, d) EtOH extracted, and (e, f) DES extracted.

To elucidate the potential mechanism of ultrasonic during the extraction process and to investigate the synergistic effect of ultrasonic-assisted DES on CGA extraction efficiency, the surface microstructure of dandelion samples before and after extraction was examined using a SEM (Fig. 5a-5f). It aimed to investigate the underlying effects of ultrasonic-assisted and various solvent combinations on extraction efficiency from a structural perspective. The crude dandelion powder indicated a relatively complete and dense structure, with a smooth and hypertrophied surface. This suggests that untreated dandelion cells and tissues maintain their cell walls and other structures, with CGA encapsulated within the cells and limited mass-transfer channels, which inhibits solubilization (Fig. 5a and 5b). Furthermore, the surfaces of the dandelion samples after EtOH solvent sonication became rough and broken, resulting in additional pores and irregular structures. This phenomenon was attributed to the cavitation effect and mechanical vibrations generated by ultrasonic waves, which compromised the integrity of the cell walls and morphology of the dandelion, increasing the mass transfer surface area and channels, facilitating the solubilization of CGA (Fig. 5c and 5d) [19,60].

Compared to the EtOH solvent ultrasonication, the dandelion samples of the DES solvent ultrasonication had porous, teardrop, and honeycomb-like loose structure on the surface, with significantly higher damage and disruption of their cells and tissues (Fig. 5e and 5f). This indicates that the combination of DES solvent and ultrasonics more efficiently degraded the microstructure of dandelion, effectively disintegrating the cell wall and other barriers, significantly increasing the surface area and pathways for mass transfer, promoting rapid release of CGA and its dissolution into the DES solvent, leading to a comparatively higher extraction efficiency [61]. At the microscopic level, DES solvent disrupted cell structures more effectively after ultrasonication, positively influencing the CGA extraction yield.

### 3.5 Adsorptive purification of CGA from dandelion

**Fig. 6:**
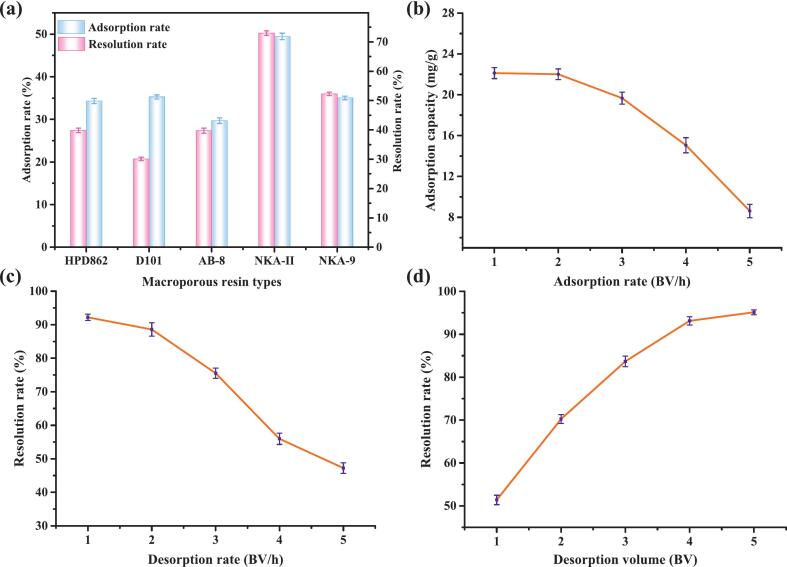
(a) Effect of different macroporous resins on the extraction yield of CGA. (b) Effect of adsorption rate on adsorption capacity. (c) Effect of desorption rate on resolution rate. (d) Effect of desorption volume on the resolution rate.

The literature suggests that CGA can be adsorbed and separated using various macroporous resins, which is influenced by the extract's characteristics and the macroporous resin's physical properties [[62], [63], [64], [65]]. Polar or weakly polar resins adsorb CGA more efficiently than nonpolar resins, suggesting that CGA is a polar or weakly polar molecule, consistent with the principle of polarity matching. Fig. 6a depicts the results of assessing the adsorption efficacy of five varieties of macroporous resins on PECGA. Furthermore, it has been observed that the adsorption rates of CGA by the polar resins NKA-II and NKA-9 were higher than those of the medium-polarity resin HPD862, the weak-polarity resin D101, and the non-polar resin AB-8 [64]. NKA-II has a greater specific surface area and demonstrates better performance, rendering it appropriate for the extraction of CGA from dandelions.

This study demonstrated that the adsorption capacity diminishes as the adsorption rate escalates (Fig. 6b). This phenomenon occurs because elevated flow rates reduce the residence time of CECGA in the resin column, resulting in insufficient time for CGA molecules to diffuse into the resin pores and adhere to the adsorption sites, therefore diminishing adsorption efficacy [66]. Moreover, when the flow rate is 2 BV/h, both the adsorption efficiency and cost-effectiveness are optimal. Moreover, at a rate of 1 BV/h, adsorption efficacy was higher; however, the duration of adsorption was prolonged, and the costs are excessively high. Whereas at the flow rates of 3 BV/h and higher, the adsorption capacity significantly diminishes.

Fig. 6c indicates that the resolution rate decreases as the desorption rate increases. With the increase in the flow velocity, turbulence gradually intensifies. Then, the eluent is renewed more rapidly, and fresh EtOH is continuously replenished to maintain efficient desorption. Furthermore, the desorbed CGA molecules were rapidly removed, minimizing re-adsorption. At a flow rate of 2 BV/h, the desorption rate peaked while the operation duration was moderate, establishing this as the optimal condition. Moreover, when the desorption volume increased, more EtOH could penetrate the resin pores and completely interact with the adsorbed CGA, causing the desorption rate to rise first and then level off (Fig. 6d). At 4 BV, the elution efficiency, solvent consumption, and operation time achieve an optimal balance; therefore, it was identified as the optimal elution volume.

After optimization of adsorption and purification parameters, the recovery of CGA using macroporous resin was maximized under the following conditions: NKA-II resin, an adsorption rate of 2 BV/h, a desorption rate of 2 BV/h, and a desorption volume of 4 BV. Under these conditions, the adsorption capacity of CGA reached 22.01 mg/g, with a resolution rate of 88.59 % and a purity of 86.14 %. These findings demonstrate that the optimized macroporous resin-based purification method is both efficient and practical for isolating CGA from dandelion.

### 3.6 Antioxidant activity of CGA in dandelion

**Fig. 7:**
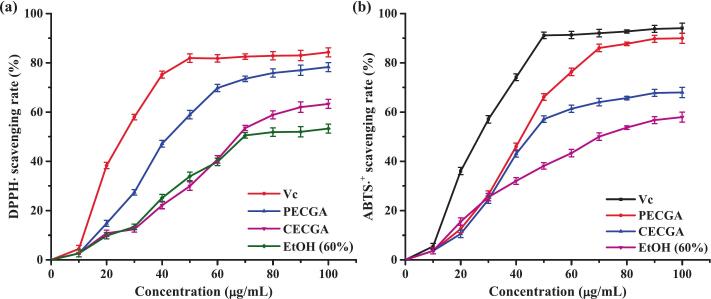
(a) DPPH and (b) ABTS radical scavenging activities of CECGA, PECGA, EtOH, and Vc.

To evaluate the antioxidant potential of dandelion extract and its applicability, two in vitro experiments were conducted: the DPPH and ABTS free radical scavenging assays. Furthermore, the concentration–response curves for Vc, PECGA, CECGA, and EtOH-extracted crude dandelion samples were compared (Fig. 7a and 7b). A significant dose-dependent enhancement in the clearance of DPPH∙ and ABTS^+^ was observed for both PECGA and CECGA. PECGA exhibited significantly improved performance compared to CECGA across all evaluated concentrations, verifying that the purification process efficiently removes co-extracted contaminants and concentrates the active ingredient. However, the clearance capacity of PECGA and CECGA remained slightly lower than that of Vc, a positive control. Moreover, at an 80 µg/mL concentration, PECGA cleared approximately 90 % of Vc, suggesting that it is only about 10 % lower than Vc. Dandelion has other antioxidant constituents than CGA, like flavonoids; however, these are predominantly eliminated during purification, resulting in minimal contributions to the overall antioxidant efficacy [67,68]. Thus, the strong antioxidant properties of PECGA can be primarily attributed to the retained fraction of CGA. Altogether, these results demonstrate that ultrasonic-assisted extraction of CGA from dandelion using DES as a solvent can increase CGA yield, providing an environmentally friendly and low-toxicity alternative to conventional organic solvents for sustainable phytochemical extraction.

### 3.7 Antibacterial properties of dandelion CGA

#### 3.7.1 Effect of PECGA from dandelion on MIC of various bacteria

**Fig. 8:**
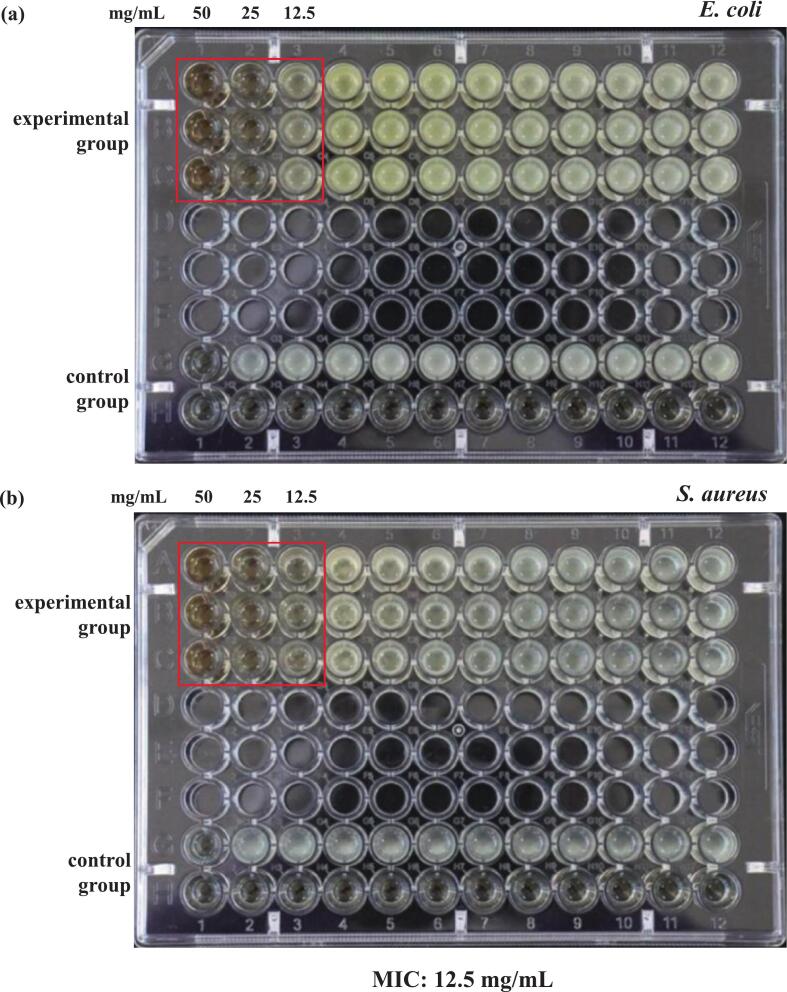
MIC test image for (a) *E. coli* and (b) *S. aureus*.

The antibacterial property is a fundamental criterion for evaluating the biological activity of materials; therefore, it holds significant importance in the research and development of novel antibacterial agents and the enhancement of industrial auxiliaries. Its efficacy and safety directly influence the clinical applicability and market viability of the products. Therefore, this study investigated the antibacterial capacity of PECGA by assessing MIC, which showed that the MIC values of PECGA in *E. coli* and *S. aureus* were 12.5 mg/mL (Fig. 8a and 8b). Moreover, when PECGA is extracted using DES as a solvent combined with an ultrasonic-assisted extraction method, it can effectively inhibit the growth of *E. coli* and *S. aureus* at lower concentrations, exhibiting good antibacterial activity. These data provide experimental evidence for its application as a natural antimicrobial agent in pharmaceutics and food preservation [69].

#### 3.7.2 SEM images of the effect of PECGA on *E. Coli* and *S. Aureus*

**Fig. 9:**
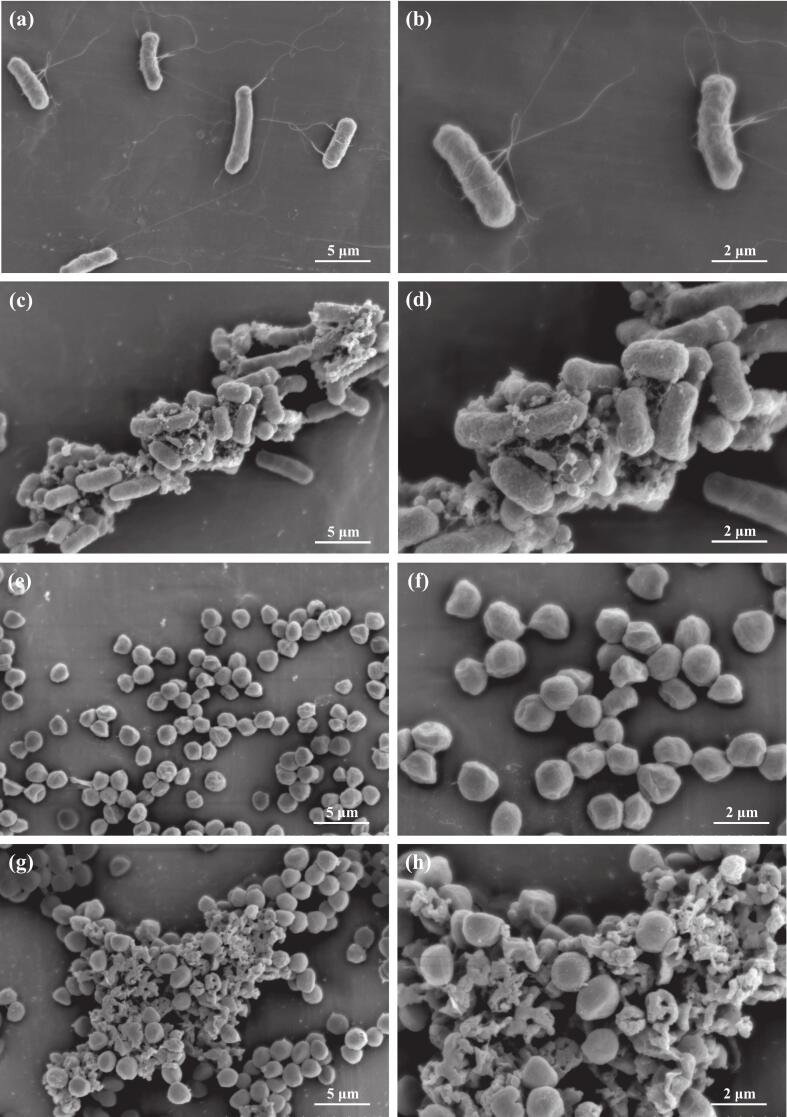
SEM images of *E. coli* (a, b) before and (c, d) after PECGA treatment, as well as *S. aureus* (e, f) before and (g, h) after PECGA treatment.

To further investigate the antibacterial properties of PECGA against *E. coli* and *S. aureus*, SEM analysis was carried out. In the blank control group (Fig. 9a and 9b), *E. coli* was observed as short rods with bluntly rounded ends, a smooth surface, and intact cell wall and membrane structures. The bacteria primarily existed as individual cells or in pairs. However, after treatment with 1.0 × MIC (12.5 mg/mL) PECGA (Fig. 9c and 9d), the bacteriophage appeared clumped, with a rough cell surface, and a damaged cell. This suggests that PECGA inhibits the normal physiological metabolism of bacteria by disrupting the integrity of the cell wall or membrane, interfering with intracellular metabolic activities, and potentially inducing oxidative stress [[70], [71], [72]].

The blank control group of *S. aureus* had a spherical shape, with a smooth surface, intact cell wall and membrane, mostly arranged in single or grape bunches (Fig. 9e and 9f). However, after treatment with PECGA (1.0 × MIC; 12.5 mg/mL), the cell wall indicated significant breakage, leakage of cellular contents, and compromised cell membrane function (Fig. 9g and 9h), which inhibited the cell from performing normal material transport and energy conversion. Due to cell wall breakage and increased adhesion, the fungus appears to clump. These findings demonstrate that PECGA can efficiently impair the structural integrity of the cell wall and membrane of *S. aureus*, thus inhibiting its growth and metabolic functions [73]. PECGA may exert its antimicrobial effects by interfering with cell wall synthesis, disrupting cell membrane function, or inducing oxidative stress [73,74]. Taken together, PECGA has significant antimicrobial activity against *E. coli* and *S. aureus*, and its antimicrobial mechanisms can help develop new natural antimicrobial agents and offer alternative strategies against antibiotic resistance.

## 4 Conclusion

In summary, this study extracted CGA from dandelion using deep eutectic solvent ultrasonic-assisted extraction. The data revealed that of the 20 DES investigated, DES-1 (bet-Ox), prepared from betaine and oxalic acid, was the most suitable extraction solvent due to its high extraction yield. The single-factor experiment validated the optimized extraction process conditions using RSM, and the extraction yield of CGA reached 4.275 mg/g. Furthermore, SEM analysis showed that the dandelion samples treated with DES-1 solvent ultrasonication were porous, honeycomb-like, and had loose surface structure, with more significant damage and destruction of cells and tissues, further improving the extraction efficiency. Moreover, after purification with macroporous resin, PECGA indicated substantial antioxidant and antibacterial activities. The UAE-DES process operates under mild conditions, allows solvent recyclability, and can be readily scaled for continuous extraction. These advantages underscore its potential as a green, efficient, and eco-friendly strategy for CGA extraction. Overall, this work provides a sustainable approach for large-scale recovery of CGA from dandelion and supports its further application in the food, pharmaceutical, and related industries.

Ethical approval

Not applicable. This study does not involve human or animal subjects.

Originality statement

This manuscript is original, has not been published, and is not under consideration for publication elsewhere, in whole or in part.

Copyright agreement

The authors agree to transfer publication rights to Elsevier B.V. upon acceptance, under the journal's copyright policy.

Funding statement

This work was partly supported by the Qinba Biological Resources and Ecological Environment Provincial and Ministerial Co-Construction National Key Laboratory (Cultivation) “City-School Co-Construction” Scientific Research Special Project (SXC-2110), and the Shaanxi Provincial Key Research and Development Projects-General Project-Agriculture (2024NC-YBXM-126). The funders had no role in study design, data collection and analysis, decision to publish, or preparation of the manuscript.

## CRediT authorship contribution statement

**Junhai Liu:** Writing – review & editing, Writing – original draft, Visualization, Project administration, Methodology, Investigation, Funding acquisition, Conceptualization. **Huan Duan:** Methodology, Investigation, Formal analysis, Data curation. **Hansheng Wang:** Validation, Investigation, Data curation. **Qi Gao:** Validation, Supervision, Data curation. **Lun Liu:** Formal analysis, Data curation. **Yinku Liang:** Validation, Data curation. **Min He:** Supervision, Data curation. **Le Xu:** Supervision, Investigation. **Xiaosha Guo:** Supervision.

## Declaration of competing interest

The authors declare that they have no known competing financial interests or personal relationships that could have appeared to influence the work reported in this paper.
