# Supplementary material for: Deep eutectic solvent extraction of chlorogenic acid from dandelion with ultrasonic-assisted: Process optimization, purification, and bioactivity
Source: Ultrason Sonochem. 2025 Sep 27;122:107579. doi: 10.1016/j.ultsonch.2025.107579 (PMC12524175; doi:10.1016/j.ultsonch.2025.107579)
Supplement: Supplementary Data 1 [file mmc1.docx]

**Supporting File**

**Deep eutectic solvent extraction of chlorogenic acid from dandelion with ultrasonic-assisted: Process optimization, purification, and bioactivity**

*Junhai Liu^a,^* ^[[1]](#footnote-1)^*, *Huan Duan^b^*, *Hansheng Wang^c^*, *Qi Gao^b^*, *Lun Liu^c^*, *Yinku Liang^a^*, *Min He^a^*, *Le Xu^c^*, *Xiaosha Guo^a^*

*^a^**Qinba State Key Laboratory of Biological Resources and Ecological Environment (Incubation)*, *School of Chemistry and Environment Science*, *Shaanxi University of Technology*, *Hanzhong 723001*, *P*.*R*. *China*

*^b^Hanzhong Shimen solid waste disposal Co., Ltd, Hanzhong 723001, P.R. China*

*^c^Hanzhong Natural Gas Investment and Development Co., Ltd, Hanzhong 723001, P.R. China*

**Table S1** Factors and levels of the experiment design

| **Level** | **A** | **B** | **C** | **D** | **E** |
| --- | --- | --- | --- | --- | --- |
| -2 | 10 | 100 | 50 | 10 | 1:10 |
| -1 | 20 | 200 | 60 | 20 | 1:20 |
| 0 | 30 | 300 | 70 | 30 | 1:30 |
| 1 | 40 | 400 | 80 | 40 | 1:40 |
| 2 | 50 | 500 | 90 | 50 | 1:50 |

**Table S2** Variance analysis of factors

| Factors | Degrees of freedom | Sum of squares of deviation | Mean square deviation | F | Pr>F |
| --- | --- | --- | --- | --- | --- |
| A | 6 | 0.7889 | 0.1315 | 18.28 | <0.0001 |
| B | 6 | 1.2943 | 0.2157 | 30.00 | <0.0001 |
| C | 6 | 1.4119 | 0.2353 | 32.72 | <0.0001 |
| D | 6 | 0.5101 | 0.0850 | 11.82 | <0.0001 |
| E | 6 | 1.0948 | 0.1825 | 25.37 | <0.0001 |

**Table S3** Comparison of extraction yield of chlorogenic acid from different plants under different extraction Methods.

| Num | Plant Material | Extraction Method and Solvents | Yield of CGA (mg/g) | Ref. |
| --- | --- | --- | --- | --- |
| 1 | *Hippophae rhamnoides* | ultrasound-assisted extraction  deep eutectic solvents | 2.45 | [1] |
| 2 | *Eucommia ulmoides* leaves | DES-based extracts | 32.45 | [2] |
| 3 | *sunflower disks* | ultrasound-assisted extraction deep eutectic solvents | 6.16 | [3] |
| 4 | *Young Fig Fruits* | deep eutectic solvents | 2.720-7.980 | [4] |
| 5 | *herba artemisiae scopariae* | natural deep eutectic solvents | 3.77 | [5] |
| 6 | *capillary artemisia* | ultrasonic-assisted extraction | 62.83 | [6] |
| 7 | *Taraxacum mongolicum* | ultrasonic-assisted extraction  70% methanol-water | 1.34 | [7] |
| 8 | *Taraxacum mongolicum* | ultrasound-assisted extraction  deep eutectic solvents | 4.27 | **This work** |

References

[1] I. Saddique, S. Akram, S. Rubab, A. Sadiqa, A. Raza, M. Mushtaq, M.A. Ghauri, Deep eutectic solvent strategy for green extraction of chlorogenic acid from sea buckthorn: optimization and sustainability, Future Journal of Pharmaceutical Sciences, 10 (2024) 120.

[2] Z. Liu, W. Ma, B. Chen, H. Pan, H. Wu, Q. Zhang, Recovery of chlorogenic acid from the DES-based extract of Eucommia ulmoides leaves by molecularly imprinted solid-phase extraction, Industrial Crops and Products, 195 (2023) 116406.

[3] J. Wu, M. Su, A. Hu, H. Wang, Extraction and recovery of chlorogenic acid from sunflower disks using a high‐efficiency system composed of deep eutectic solvents and macroporous resins, Journal of Food Processing and Preservation, 46 (2022) e16856.

[4] Q. Zhang, Y. Peng, Y. Xu, F. Li, S. Liu, D. Bukvicki, Q. Zhang, S. Lin, M. Wang, T. Zhang, D. Wu, W. Qin, Extraction, Characterization, and In Vitro Biological Activity of Polyphenols from Discarded Young Fig Fruits Based on Deep Eutectic Solvents, Antioxidants, 13 (2024) 1084.

[5] Y. Yue, Q. Huang, Y. Fu, J. Chang, A quick selection of natural deep eutectic solvents for the extraction of chlorogenic acid from herba artemisiae scopariae, RSC advances, 10 (2020) 23403-23409.

[6] Y. Dai, K.H. Row, Ultrasonic-Assisted Extraction of Chlorogenic Acid from Capillary Artemisia with Natural Deep Eutectic Solvent-Functionalized Cellulose, Analytical Letters, 54 (2021) 1840-1857.

[7] P. Xu, X. Xu, A. Khan, T. Fotina, S. Wang, Antibiofilm activity against Staphylococcus aureus and content analysis of Taraxacum Officinale phenolic extract, Polish journal of veterinary sciences, (2021) 243-251-243-251.

1. * Corresponding author. Tel: +86 916 2641660.

   *E-mail address*: iamliujunhai@126.com (J. Liu). [↑](#footnote-ref-1)
